# Coronary blood flow in heart failure: cause, consequence and bystander

**DOI:** 10.1007/s00395-022-00909-8

**Published:** 2022-01-13

**Authors:** Gerd Heusch

**Affiliations:** Institute for Pathophysiology, West German Heart and Vascular Center, University of Essen Medical School, University of Duisburg-Essen, Hufelandstr. 55, 45147 Essen, Germany

**Keywords:** Coronary blood flow, Coronary microcirculation, Coronary reserve, Extravascular compression, Heart failure

## Abstract

Heart failure is a clinical syndrome where cardiac output is not sufficient to sustain adequate perfusion and normal bodily functions, initially during exercise and in more severe forms also at rest. The two most frequent forms are heart failure of ischemic origin and of non-ischemic origin. In heart failure of ischemic origin, reduced coronary blood flow is causal to cardiac contractile dysfunction, and this is true for stunned and hibernating myocardium, coronary microembolization, myocardial infarction and post-infarct remodeling, possibly also for the takotsubo syndrome. The most frequent form of non-ischemic heart failure is dilated cardiomyopathy, caused by genetic mutations, myocarditis, toxic agents or sustained tachyarrhythmias, where alterations in coronary blood flow result from and contribute to cardiac contractile dysfunction. Hypertrophic cardiomyopathy is caused by genetic mutations but can also result from increased pressure and volume overload (hypertension, valve disease). Heart failure with preserved ejection fraction is characterized by pronounced coronary microvascular dysfunction, the causal contribution of which is however not clear. The present review characterizes the alterations of coronary blood flow which are causes or consequences of heart failure in its different manifestations. Apart from any potentially accompanying coronary atherosclerosis, all heart failure entities share common features of impaired coronary blood flow, but to a different extent: enhanced extravascular compression, impaired nitric oxide-mediated, endothelium-dependent vasodilation and enhanced vasoconstriction to mediators of neurohumoral activation. Impaired coronary blood flow contributes to the progression of heart failure and is thus a valid target for established and novel treatment regimens.

## Introduction

**Fig. 1:**
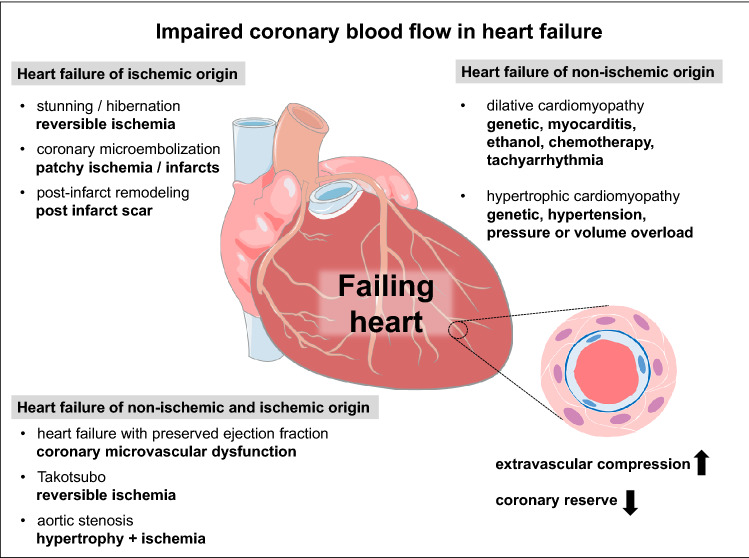
Impairment of coronary blood flow in heart failure of ischemic origin, of non-ischemic origin and of a pathogenesis with ischemic and non-ischemic contributions

Heart failure and atherosclerosis are frequent and frequently co-exist, as they develop not only from more or less specific genetic predispositions but also from life style-related risk factors and comorbidities, such as physical inactivity, obesity [201] and metabolic syndrome, diabetes, hypertension, but also from environmental pollution [187]. The co-existence of predisposing risk factors and comorbidities, coronary atherosclerosis and coronary microvascular dysfunction is particularly obvious in patients who have heart failure with preserved ejection fraction. The interaction between coronary atherosclerosis and heart failure is complex. Coronary atherosclerosis on the one hand can induce myocardial ischemia and infarction which then causes heart failure. On the other hand, genetic mutations can cause heart failure, and coronary blood flow even in the absence of coronary atherosclerosis is then impaired as a consequence of heart failure. Then, both heart failure and impaired coronary blood flow impact on each other—any form of heart failure predisposes to myocardial ischemia through increased extravascular compression and increased coronary vasoconstriction in response to neurohumoral activation, and any form of myocardial ischemia further impairs left ventricular (LV) function (Fig. 1). The coronary circulation in heart failure is characterized by morphological alterations (arteriolar hypertrophy, capillary rarefication) and functional abnormalities, such as impaired endothelium-dependent and metabolic vasodilation, enhanced vasoconstriction to mediators of neurohumoral activation, and increased extravascular compression. A positive interaction between heart failure and impaired coronary vascular function, as evidenced by reduced coronary dilator reserve in heart failure, predisposes to poor clinical outcome. Comprehensive review articles on the coronary circulation in more general [12, 45, 74, 183], the coronary microcirculation in more particular, [40, 170, 273] and on the coronary circulation in specific forms of heart failure, e.g., hypertrophy, [10, 26, 31] heart failure of hypertensive origin [263] or heart failure with preserved ejection fraction [175, 216, 239] already exist. The present review attempts a comprehensive analysis of the common features of coronary blood flow impairment in the entire spectrum of heart failure syndromes and the cause-and-consequence relationships between heart failure and coronary blood flow. More specifically, this review identifies the common grounds of impaired coronary dilator reserve in all heart failure as well as the more specific defects of the coronary circulation in the different heart failure entities.

## Heart failure of ischemic origin

A reduction in cardiac contractile function is the earliest and most obvious manifestation of each critical reduction in coronary blood flow, whether reversible or not. The critical threshold of coronary blood flow is 8–10 µl per g of myocardial tissue and cardiac cycle [91].

### Stunned and hibernating myocardium

Stunned and hibernating myocardium are characterized by reversible contractile dysfunction during the recovery from an episode of myocardial ischemia (stunning) or during still ongoing more moderate myocardial ischemia (hibernation) [94]. Heart failure can develop from such reversible contractile dysfunction acutely (stunning) or more chronically (hibernation), provided the respective coronary perfusion territory, the severity and the duration of coronary blood flow reduction are large enough [94].

*Stunned myocardium* Almost by definition, stunned myocardium is reperfused, i.e., in chronically instrumented conscious dogs, there is typically an initial reactive hyperemia followed by a normalization of myocardial blood flow over several hours during which some transmural redistribution of blood flow at the expense of subendocardial layers remains [99, 100]. Depending on the severity and duration of the preceding myocardial ischemia, full recovery of regional contractile function occurs over several hours to days [100, 140, 236]. When reperfusion occurs through a residual stenosis in chronically instrumented conscious pigs, coronary perfusion abnormalities and contractile dysfunction persist longer and may induce hibernation [238]. Whereas the myocardial contractile dysfunction of stunned myocardium is caused by increased reactive oxygen species formation and impaired excitation–contraction coupling, [94] there is also a coronary vascular stunning component, with an impaired reactive hyperemia response after brief coronary occlusion [247] and impaired vasodilator responses to intravenous adenosine or papaverine, [19] and a particularly impaired endothelium-dependent coronary vasodilator response to acetylcholine [47] in anesthetized dogs and pigs [141]. Whereas the Gregg phenomenon (an increase in contractile function in response to increased coronary blood flow) is not operative in normal myocardium, [207] the coronary autoregulation in stunned myocardium appears blunted, predisposing it to a Gregg effect, i.e., there is increased regional contractile function in anesthetized dogs to intravenous dipyridamole or papaverine [223] and in anesthetized pigs to intracoronary adenosine [208].

**Fig. 2:**
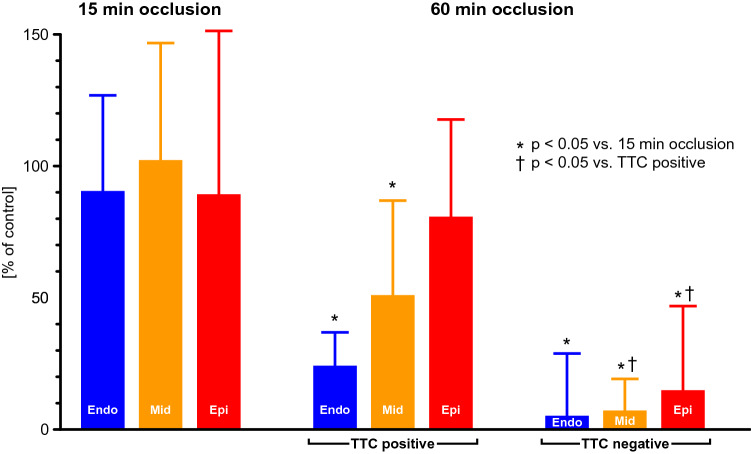
Increment in coronary blood flow in response to intracoronary acetylcholine (in % of dilator response at baseline) at 30 min reperfusion following either 15 min or 60 min coronary occlusion in anesthetized dogs, in reversibly (TTC-positive) and irreversibly (TTC-negative) injured myocardium. Endo: flow to subendocardial layers; Mid: flow to midmyocardial layers; Epi: flow to subepicardial layers. TTC, triphenyl tetrazolium chloride. From [47] by permission

Most importantly, stunning contributes to contractile dysfunction following non-transmural myocardial infarction, i.e., there is both an irreversible and a reversible component of contractile dysfunction. In anesthetized dogs with 2 h coronary occlusion, regional myocardial blood flow recovered to 50% of baseline after 2 h reperfusion and regional contractile function recovered back to about 40% of baseline within 2 weeks. [50] In conscious dogs, which were otherwise healthy and without coronary atherosclerosis, 1 h coronary occlusion induced severe regional contractile dysfunction which recovered back to > 50% within 4 weeks, but there was no recovery after 3 h coronary occlusion. [129] In anesthetized dogs, the coronary dilator response to intracoronary acetylcholine was severely impaired at 30 min reperfusion in the myocardium surviving 1 h coronary occlusion, particularly in its subendocardial layers (Fig. 2) [47].

*Stunning in the clinic* Pure stunning, i.e., fully reversible contractile dysfunction following an episode of myocardial ischemia, occurs clinically following percutaneous coronary intervention (PCI) [102, 161, 211] or a protocol of exercise-induced ischemia [4, 61, 135] but rarely poses a clinical problem, notably does not cause heart failure per se. [87] However, stunning may contribute to other myocardial ischemia-related heart failure scenarios, e.g., recovery from myocardial infarction (see above [24, 210, 240]) or from cardioplegic ischemic cardiac arrest. Unfortunately, sequential measurements of coronary blood flow and its relation to contractile function during the recovery from myocardial infarction or cardioplegia are not available. There is also vascular stunning, a reduced coronary vasodilator response to dipyridamole in patients after PCI, [252] but its functional importance is not really clear.

*Hibernating myocardium* Different from stunning with its transient nature, hibernation is a sustained state of regional myocardial contractile dysfunction which may indeed cause chronic heart failure. By definition, hibernating myocardium has reduced blood flow and its contractile dysfunction recovers after revascularization [22, 88, 94, 98, 118, 185, 186]. Hibernation was originally regarded as an adaptive response of the myocardium to ischemia, in that contractile function was downregulated to match the decrease in myocardial blood flow such that the myocardium could retain its viability and contractile function recover after revascularization [186]. Indeed, evidence for such perfusion–contraction matching not only during brief episodes of myocardial ischemia [14] was provided in a number of experimental studies in anesthetized and chronically instrumented conscious dogs and pigs, and the adaptive nature of such perfusion–contraction matching was supported by the recovery of metabolic perturbations during the progression from early to more sustained ischemia over several hours [94, 196]. The idea of an adaptive downregulation in response to reduced blood flow in hibernating myocardium was challenged since in some experimental studies, in chronically instrumented conscious pigs with coronary stenosis, contractile function was reduced but myocardial blood flow was not [212, 213]. A heated debate on whether hibernating myocardium was an adaptation to persistent ischemia or a result of repetitive stunning followed, but resolved by elegant experiments of Canty and colleagues who demonstrated in chronically instrumented conscious pigs with coronary stenosis, that indeed there is a progression from repetitive stunning to hibernation where myocardial blood flow and coronary reserve are reduced [55, 57]. When such chronic hibernating myocardium with reduced regional contractile function and blood flow affects both the left anterior descending and the left circumflex coronary arteries in pigs, a typical situation of compensated heart failure develops [56]. Hibernation characterized not only contractile function and metabolism distal to a chronic coronary stenosis, but also the coronary circulation which developed atrophy of larger (> 75 µm diameter) and hypertrophy of smaller (< 75 µm diameter) microvessels distal to the stenosis [148]. Induction of angiogenesis by endothelial nitric oxide synthase transfection in a pig model of hibernation, conversely, improved blood flow and contractile reserve [125]. Revascularization of chronically hibernating myocardium quickly normalizes adenosine-recruitable coronary reserve but recovery of contractile function is more delayed [171].

*Hibernating myocardium in the clinic* In patients with chronic coronary artery disease and contractile dysfunction, there is solid evidence from studies using positron emission tomography (PET) that myocardial blood flow in the hibernating regions is reduced [88, 98, 258] but higher than in regions which did not recover contractile function after revascularization [41, 276]. Dipyridamole-recruited coronary reserve is more reduced in patients with coronary artery disease and LV dysfunction than in those without LV dysfunction [256]. The viability of hibernating myocardium which is then an indication for revascularization is best assessed by a combination of imaging of decreased myocardial blood flow and increased glucose uptake by PET [69].

Whereas the prognostic benefit from optimal medical therapy vs. that from revascularization in patients with stable coronary artery disease and angina is contentious, [18, 137] it is particularly the group of patients with coronary artery disease and ischemic heart failure who benefit from coronary revascularization. In the STICH trial, 1212 patients with chronic coronary artery disease and a LV ejection fraction of ≤ 35% were randomized to medical treatment of surgical revascularization, and those with revascularization had better outcome in mortality, cardiovascular mortality and hospitalization for heart failure, [104, 259] notwithstanding some critical considerations on the value of viability testing in this trial [5]. Also, in the otherwise neutral large ISCHEMIA trial, in 5179 patients with stable coronary artery disease and angina, it was the subgroup of 398 patients with a history of heart failure or LV ejection fraction ≥ 35 but < 45% who had a worse 4-year outcome than patients without heart failure or LV dysfunction. Of note, however, this subgroup of patients had a better outcome in terms of all-cause mortality, cardiovascular mortality or hospitalization for heart failure with coronary revascularization by PCI or coronary artery bypass graft surgery than with medical therapy [131]. Although pre-specified, this was a subgroup analysis only and must be considered hypothesis-generating at this point. However, it does support the notion that coronary revascularization is of particular benefit for patients with heart failure of ischemic origin, supporting the concept of hibernating myocardium [94].

### Coronary microembolization

Coronary microembolization occurs spontaneously or iatrogenically during PCI when atherothrombotic particulate debris and soluble vasoconstrictor, thrombogenic and inflammatory substances are released from erosion or rupture of an atherosclerotic plaque [117]. Spontaneous coronary microembolization may be clinically silent and become only apparent by chance in elevated serum troponin concentrations. Direct evidence for coronary microembolization is achieved only when it occurs clinically as an acute coronary syndrome or during PCI [117]. Repetitive, also repetitive clinically silent coronary microembolization may ultimately result in diffuse ischemic cardiomyopathy [117].

*In animal experiments*, coronary microembolization of inert particles was historically used to induce acute heart failure and cardiogenic shock [2]. Franciosa et al. then introduced the intracoronary embolization of glass beads of 400–600 µm in diameter into conscious dogs as a model of chronic heart failure, [62] which was subsequently further refined by Sabbah et al. who used repeated intracoronary injections of polystyrene microspheres of 70–110 µm in diameter to induce a stable situation of chronic heart failure in conscious dogs [199]. This heart failure model is characterized by LV hypertrophy, patchy myocardial fibrosis, and neurohumoral activation, [198, 199] and such model was also replicated in sheep [107] and pigs [228]. The microembolization-induced heart failure model has the advantage of reasonable stability such that therapeutic strategies can be studied. Using this model, different treatment regimens, including metoprolol, enalapril [198] and cell therapy [228] were tested. With a more limited repetitive coronary microembolization using microspheres of 115 µm in diameter in conscious dogs, a situation of heart failure with preserved ejection fraction, no reduction in end-systolic elastance and in ventricular relaxation but with intravascular volume expansion, neurohumoral activation and elevated LV end-diastolic pressure was induced [81]. Somewhat surprisingly, most of these studies which intentionally impaired coronary blood flow to induce heart failure did not report coronary blood flow at baseline before and after repetitive coronary microembolization and established heart failure. More acutely, coronary microembolization is typically characterized by elevated baseline coronary blood flow through reactive hyperemia in the coronary vasculature around the microembolized vascular territory and reduced adenosine-recruitable coronary blood flow through physical obstruction of some microvessels, acting jointly to reduce the amplitude of coronary reserve; [217] the same elevation of baseline coronary blood flow and reduction of coronary reserve is seen *in patients* with peri-interventional coronary microembolization [84]. In one study with repetitive coronary microembolization in dogs, the coronary vasodilator response to intravenous acetylcholine was depressed before heart failure had developed, and adenosine-recruitable coronary reserve was decreased with established heart failure [120].

### Myocardial infarction and post-infarct remodeling

Myocardial infarction results from sustained and severe impairment of coronary blood flow after rupture or erosion of an epicardial coronary atherosclerotic plaque and/or coronary microvascular obstruction and manifests in injury to the myocardium and the coronary microcirculation; reperfusion is mandatory to salvage myocardium from impending infarction but inflicts additional injury to the myocardium and the coronary microcirculation [93]. Heart failure can result from myocardial infarction acutely in the form of cardiogenic shock or more chronically as a consequence of LV remodeling [97]. Since myocardial infarction affects a particular coronary perfusion territory, distinction is needed between blood flow to the infarcted and to the non-infarcted remote myocardium.

*The infarct region* The coronary circulation experiences massive injury during myocardial ischemia and in the following reperfusion, including increased vascular permeability and edema formation, platelet and leukocyte plugging and ultimately capillary destruction and intra-myocardial hemorrhage [16, 93]. In its extreme form, this coronary microvascular injury manifests during reperfusion following myocardial ischemia in the form of coronary microvascular obstruction and a no-reflow phenomenon, in both experimental animals and patients with reperfused acute myocardial infarction [92]. In experimental studies, coronary microvascular obstruction is best quantified by lack of endothelial staining with thioflavin, and in preclinical and clinical studies, it is quantified as an increased microvascular resistance by measurement of perfusion pressure and coronary blood flow or visualized by magnetic resonance imaging (MRI) (see Figs. 2 and 3 in [92]). In the further time course after acute myocardial infarction, not only the myocardium remodels and, if the infarcted region is large enough, eventually develops heart failure, [97, 142, 168, 180] but also the culprit coronary circulation remodels. Following the microvascular injury and destruction during immediate reperfusion, there is infarct healing with coronary angiogenesis and myocardial revascularization, and the disruption of angiogenesis contributes to the development of post-myocardial infarct heart failure in mice [215]. The post-infarct myocardial revascularization is dependent on angiogenic factors, notably vascular endothelial growth factor (VEGF), [15, 193] which in turn is increased by paracrine mechanisms involving cardiomyocyte alpha 1 receptor activation [279] and beta blockade in rats, [193] and nitric oxide in mice, which again is promoted by statins [128] or cell therapeutic approaches [112, 127]. Stimulation of angiogenesis in experimental animals improves LV function and attenuates the development of heart failure. [15, 128, 193, 215, 266] There appears to be a positive feed-back vicious cycle between heart failure following myocardial infarction and an inflammatory dysregulation of the bone marrow niche to mobilize cells for myocardial or coronary vascular repair in mice and also humans [101].

**Fig. 3:**
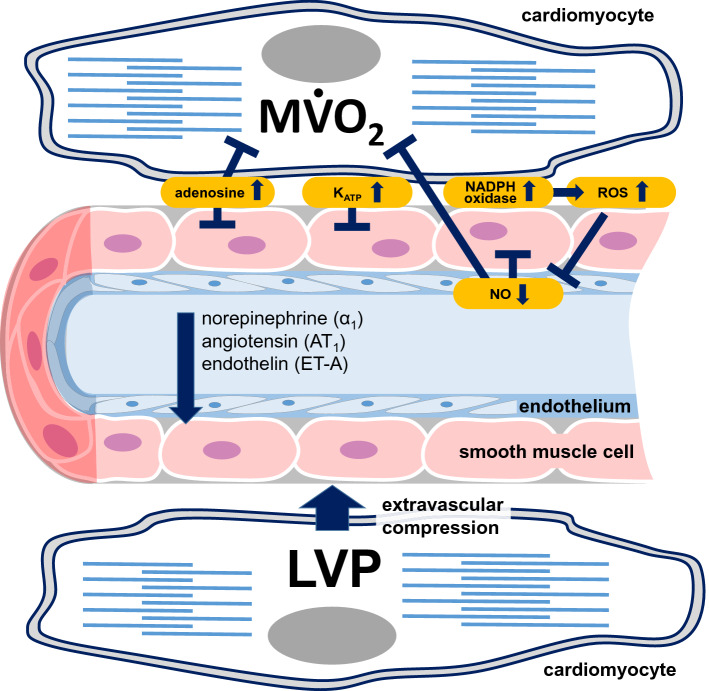
Mechanisms of impairment of coronary blood flow in heart failure: mechanical extravascular compression by left ventricular pressure (LVP), attenuated metabolic and nitric oxide (NO)-mediated endothelium-dependent coronary vasodilation secondary to increased formation of reactive oxygen species (ROS) formation, increased vasoconstriction to mediators of neurohumoral activation (norepinephrine, angiotensin, endothelin)

*The remote region* In experimental studies, alterations in coronary blood flow were also seen in the non-infarcted remote myocardium. In pigs with left circumflex coronary artery occlusion, cardiac output 2–3 weeks later was reduced and there was neurohumoral activation with increased plasma norepinephrine, epinephrine, angiotensin, and endothelin, reflecting LV dysfunction [77]. In this model, exercise-induced coronary vasodilation was preserved but attenuated, [77] and increased activation of ATP-dependent K channels, [147] maintenance of nitric oxide-mediated endothelium-dependent vasodilation [78] and attenuated vasoconstrictor impact of angiotensin [145] and endothelin [146] contributed to such adaptation of the remote coronary circulation in post-infarct left ventricular dysfunction [43]. These studies used systemic blockers to address the mediator mechanisms; it is therefore unclear, in which cellular compartment (myocardial [277] vs. vascular) the activation of ATP-dependent K channels occurs.

*Coronary microvascular obstruction in the clinic* Microvascular obstruction occurs in many patients with successfully reperfused myocardial obstruction, ranging from 5 to 70% depending on the method and parameter and the time of its assessment. [92] Not only infarct size but also the extent of coronary microvascular obstruction on MRI is a major determinant of cardiogenic shock [190]. However, coronary microvascular obstruction after successful reperfusion by PCI also predicts the long-term development of LV dysfunction [20, 134, 250] and clinical outcome in terms of mortality and hospitalization for heart failure [29, 39, 119, 191, 255, 268]. Infusion of bone marrow-derived or circulating progenitor cells into the infarct-related coronary artery in patients with reperfused myocardial infarction increased adenosine-recruitable coronary reserve on follow-up in the TOPCARE-AMI and REPAIR-AMI trials, [9, 52, 53] and this effect was associated with improved LV function and clinical outcome [8, 203]. Unfortunately, the clinical value of such autologous cell therapy approaches in patients with acute myocardial infarction remains uncertain, given the lack of a positive large prospective clinical outcome trial [139].

Clinically, in patients with uncomplicated reperfused acute myocardial infarction, adenosine-recruitable coronary velocity reserve (Doppler) is decreased immediately after PCI in the culprit and the non-culprit coronary artery as compared to propensity-matched controls. [38] The impairment in coronary reserve of the non-culprit coronary arteries as measured by PET is more severe in patients with coronary artery disease and heart failure than in those without heart failure [253]. Patients with myocardial infarction in the absence of significant obstructive coronary artery disease (MINOCA) have milder impairment of coronary blood flow and coronary reserve than those with classical myocardial infarction and obstructive coronary artery disease [149] and better outcome on follow-up, including the development of heart failure; [173] however, the specific role of coronary blood flow impairment for heart failure development in MINOCA is not clear at present.

## Heart failure of non-ischemic origin

### Dilative cardiomyopathy

**Fig. 4:**
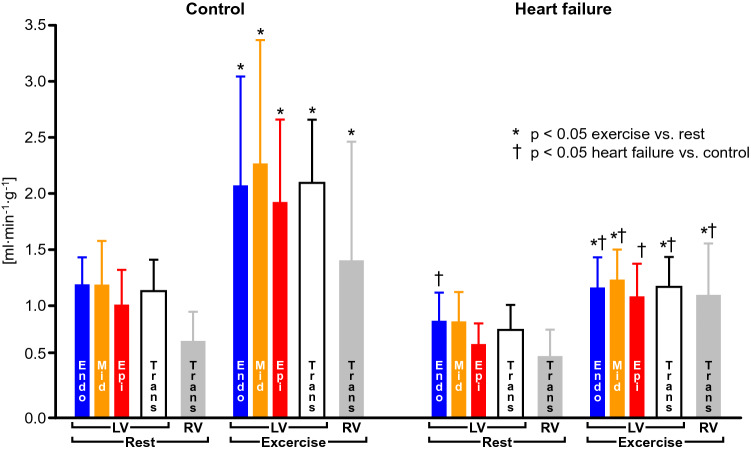
Attenuated increases in regional myocardial blood flow of the left (LV) and right (RV) ventricle in chronically instrumented conscious dogs with pacing-induced heart failure during treadmill exercise. Endo: flow to subendocardial layers; Mid: flow to midmyocardial layers; Epi: flow to subepicardial layers. Trans: flow to the entire transmural region. Data from [160]

Dilated cardiomyopathy in humans arises from genetic mutations in sarcomeric or mitochondrial proteins, [195] myocarditis [249] or toxic agents, such as ethanol [59] or chemotherapy, [83, 241] and from sustained tachyarrhythmias [49, 105]. Pacing-induced heart failure in experimental animals does not only mimic the clinical syndrome of tachycardia-induced cardiomyopathy but is also considered as a model of dilated cardiomyopathy, which mimics the features of ventricular dilatation and dysfunction, systemic congestion, exercise intolerance and dyspnea, neurohumoral activation, cardiomyocyte loss and hypertrophy of remaining cardiomyocytes, fibrosis and apoptosis [90]. In conscious pigs with chronic supraventricular pacing, there is capillary rarefication, reduced myocardial blood flow, and adenosine-recruitable coronary reserve particularly in the LV subendocardium [106, 220, 221]. Reduced baseline myocardial blood flow and adenosine-recruitable coronary reserve were also seen in conscious dogs with chronic right ventricular pacing, but there was no evidence for capillary rarefication [209]. In early stages of pacing-induced heart failure, despite neurohumoral activation and increased plasma concentrations of vasoconstrictor substances (norepinephrine, angiotensin, endothelin), [123, 162] nitric oxide formation may be increased and act to preserve coronary blood flow [162, 200]. Also, ATP-dependent K-channel activation may contribute to attenuate decreases in myocardial blood flow in dogs with pacing-induced heart failure [110, 244, 269]. While endothelium-dependent coronary vasodilation is still preserved, however, adenosine-recruitable coronary vascular reserve is already reduced through increased extravascular compression [242]. In an early state of pacing-induced heart failure, the vasoconstrictor effect of angiotensin was attenuated and the bradykinin-dependent vasodilator effect of the ACE inhibitor enalapril enhanced, supporting the notion of an increased nitric oxide formation [163]. Conscious dogs with chronic left ventricular pacing and established heart failure then had decreased epicardial coronary dilation and coronary blood flow response to acetylcholine and less coronary vascular nitrite formation in response to acetylcholine ex vivo, suggesting a defect in endothelial nitric oxide formation [227, 265]. The defect of endothelial nitric oxide formation in dogs with pacing-induced heart failure also impaired the cholinergic coronary vasodilation as part of the Bezold-Jarisch or carotid chemoreflex [278]. The reduced nitric oxide formation in established pacing-induced heart failure in dogs also induced a switch in cardiac substrate utilization from free fatty acid to glucose uptake. [189] The attenuation of nitric oxide-mediated, endothelium-dependent coronary vasodilation in pacing-induced heart failure is secondary to nitric oxide inactivation by reactive oxygen species [157] and NADPH oxidase activity [231, 275]. It is currently unclear in which cellular compartment (vascular or myocardial) the responsible NADPH oxidase activation occurs and where the increased reactive oxygen species formation originates; [154] this distinction, however is important to decide whether the impaired coronary vasomotion is a consequence of heart failure (myocardial origin) or a bystander (vascular origin) induced by the conditions leading to heart failure, e.g., sustained rapid pacing (Fig. 3). In any event, increased endothelial nitric oxide synthase activity [231, 248] by statins preserves endothelium-dependent coronary vasodilation in pacing-induced heart failure. Pacing-induced heart failure, [221] endothelium-dependent coronary vasodilation, [251] and endothelial nitric oxide formation [71] recover after termination of chronic pacing over several weeks. The relatively fast recovery of the pacing-induced heart failure after cessation of pacing is a disadvantage for the study of treatment regimens in this model, but it does mimic the clinical syndrome of tachycardic cardiomyopathy particularly well [90]. In dilated cardiomyopathy of tachycardic origin, the impairment of the coronary circulation plays a particularly prominent role since tachycardia increases myocardial oxygen consumption and decreases diastolic duration, thereby increasing the susceptibility to myocardial ischemia [14, 89]. In conscious dogs [160, 245] and pigs [123] with chronic rapid pacing, the exercise-induced increases in cardiac output but also in regional myocardial blood flow to the left and right ventricle, skeletal muscle blood flow and renal blood flow were attenuated (Fig. 4). The decrease in myocardial blood flow at baseline and during exercise in dogs with chronic pacing-induced heart failure was associated with a proportionate decrease in myocardial oxygen consumption and occurred in the absence of myocardial ischemia (net lactate production) [245]. The metabolic coronary vasodilation during pacing-induced tachycardia in dogs with established pacing-induced heart failure depends on nitric oxide formation, [229] and nitric oxide formation may inhibit myocardial oxygen consumption in the failing heart [243].

The calcium antagonist amlodipine, [124] but not the angiotensin AT1 receptor antagonist valsartan [33] improved myocardial blood flow during exercise in pacing-induced heart failure. Not only extravascular compression by increased left ventricular end-diastolic pressure but also increased plasma vasoconstrictor concentrations from neurohumoral activation limit coronary blood flow in heart failure. The muscle metaboreflex-induced sympathetic activation during exercise in dogs with pacing-induced heart failure induced coronary vasoconstriction, [7] which was abrogated by alpha1-adenoceptor blockade with prazosin; [34] prazosin also attenuated resting coronary vasomotor tone in dogs with pacing-induced heart failure [232]. Endothelin-A receptor blockade also increased coronary blood flow during exercise in dogs with pacing-induced heart failure [103]. Apparently, coronary vasomotion in established pacing-induced heart failure at rest and during exercise is characterized by reduced nitric oxide-mediated, endothelium-dependent vasodilation and enhanced vasoconstriction by norepinephrine and endothelin.

In clinical dilated cardiomyopathy, impaired endothelium-dependent coronary vasodilation of the epicardial coronary arteries and of the microcirculation in response to intracoronary acetylcholine was demonstrated by angiography and Doppler velocity flow measurements (Table 1); [27, 138, 246] an impaired adenosine-recruitable coronary reserve was only apparent in patients with chronic, [27, 246] but not with acute onset—idiopathic dilated cardiopathy [138].

**Table 1:** Measurement of coronary reserve in patients with heart failure

**I. Vasodilator stimulus**
Postocclusive reactive hyperemia
Adenosine, intracoronary or intravenous
Dipyridamole, intravenous
Contrast medium, intracoronary or intravenous
**II. Imaging technique**
Angiography, invasive
Doppler flow velocity, invasive
Contrast echocardiography, non-invasive
Single photon emission computed tomography, non-invasive
Positron emission tomography, non-invasive
Nuclear magnetic resonance imaging, non-invasive

Decreased coronary reserve, as recruited by intravenous dipyridamole, was confirmed for patients with chronic idiopathic dilated cardiomyopathy using PET, [159, 224, 254] and decreased coronary reserve [159] and the spatial heterogeneity of myocardial blood flow [214] were associated with poor prognosis (mortality, heart failure progression). On MRI of patients with dilated cardiomyopathy, there was evidence for an increased extracellular matrix [111, 158] in association with reduced myocardial blood flow at rest [111] and with reduced angiographic coronary vasodilator response to intracoronary acetylcholine [158]. Somewhat surprisingly, patients with dilated cardiomyopathy had no reduction, but a modest increase in myocardial blood flow at rest, but again a decrease in adenosine-recruitable coronary reserve in MRI perfusion imaging [76]. The decrease in adenosine-recruitable coronary reserve was, however, not sufficient to induce a myocardial oxygen deficiency, supporting the non-ischemic nature of idiopathic dilated cardiomyopathy [36]. Apart from endothelial dysfunction as evidenced by the impaired coronary dilator response to acetylcholine, there is also neurohumoral activation in patients with dilated cardiomyopathy, [63] and antagonism of neurohumoral activation is an essential part of all medical treatment of heart failure [75, 108]. However, to which extent attenuated coronary vasoconstriction, as evidenced in the above experimental studies, contributes to the treatment success in patients with heart failure is unclear, given the systemic effects of such treatment on heart rate, blood pressure and ventricular function which all impact on coronary blood flow. Collectively, the clinical imaging data in patients with idiopathic dilated cardiomyopathy confirm a depression of endothelium-dependent coronary vasodilation and a reduction of coronary vasodilator reserve.

### Hypertrophic cardiomyopathy

Hypertrophy of the myocardium develops as an adaptive response to pressure or volume overload or can be the manifestation of a genetic disease [167, 264]. In both scenarios, the hypertrophy may decompensate into heart failure, with or without preserved ejection fraction [26]. Remodeling and dysfunction of the coronary microcirculation are typically involved in hypertrophic cardiomyopathy, even in the absence of atherosclerosis [26].

In experimental animals, LV hypertrophy is morphologically not only characterized by increased cardiomyocyte cross-sectional area, but also by decreased capillary density and increased intercapillary distances [10, 17]. For myocardial blood flow and its distribution, it is important to distinguish between scenarios where the coronary circulation is also exposed to pressure or volume overload (supravalvular aortic stenosis/banding, hypertension) or where coronary perfusion pressure is reduced (aortic valve stenosis). In chronically instrumented conscious dogs with banding of the ascending aorta at 6–8 weeks of age, LV myocardial blood flow as assessed by the microsphere technique was increased after hypertrophy had developed after several months and even further increased when hypertrophy had decompensated to failure, as defined by increased LV end-diastolic pressure > 18 mmHg [172]. Using the same model, again increased myocardial blood flow commensurate with the increased myocardial work was seen, and there was no depletion of myocardial energy-rich phosphates, not even when fractional shortening was decreased in dogs with decompensated hypertrophy [65]. This model is, however, characterized by decreased adenosine-recruitable coronary reserve [17, 109]. During exercise, increases in myocardial blood flow were greater in dogs with LV hypertrophy commensurate with their greater increase in myocardial oxygen consumption. For the increase in coronary blood flow during exercise, dogs with a hypertrophied heart used a greater activation of ATP-dependent K-channels than normal dogs; [144] however, the subendocardial were less than the subepicardial blood flow increases, reflecting potential susceptibility to ischemia [11, 44]. The relative underperfusion of subendocardial layers of hypertrophied myocardium during exercise was attributed to increased extravascular compression, [44] but not to a deficit in nitric oxide bioavailability [46]. Coronary blood flow returned to normal after regression of hypertrophy [109]. Different from the above studies which used supravalvular aortic banding, experimental aortic valve stenosis in young dogs also resulted in LV hypertrophy several months later, but a more substantial reduction of adenosine-recruitable coronary reserve and a subnormal increase in blood flow during pacing-induced tachycardia particularly in the subendocardium [3]. Likewise, in chronically instrumented conscious dogs with renal hypertension, LV hypertrophy developed and coronary autoregulation was impaired such that at the lower range of coronary autoregulation (40–70 mmHg), myocardial blood flow was reduced to a greater extent than in normal dogs, particularly in subendocardial layers [80]. In pigs with corticosterone-induced hypertension, LV hypertrophy developed over 12 weeks, and the dobutamine stress-recruited perfusion reserve on MRI was reduced as compared to normal pigs [192]. Volume overload by severe experimental mitral regurgitation in dogs also induced LV hypertrophy after several months [28, 274]. Myocardial blood flow at rest and its increases during pacing and intravenous adenosine were, however, not different between dogs without or with mitral regurgitation [28, 274]. Nevertheless, energy-rich phosphates [274] and contractile function [28] were impaired in these dog studies with chronic mitral regurgitation, thus excluding a role of coronary blood flow in these impairments.

Also, in patients with hypertrophic cardiomyopathy, but absence of valve disease or hypertension, there are structural alterations in the coronary circulation; at autopsy, remodeling of intramural coronary arteries (< 1500 µm in diameter) with intimal and medial hypertrophy and narrowed lumen were seen in the majority of cases [136]. Small vessel disease of intramural coronary arteries (20–1000 µm in diameter) was also evident in the autopsy of patients with hypertrophic cardiomyopathy of various origin, including hypertension, with significant luminal narrowing which correlated to measures of hypertrophy and presence of fibrosis [233]. In young patients with hypertrophic cardiomyopathy and sudden cardiac death, there was morphological evidence of small vessel coronary disease and patchy myocardial scars, supporting the occurrence of ischemia in the natural history of hypertrophic cardiomyopathy [13]. Patients with hypertrophic cardiomyopathy in the absence of other cardiovascular disease, notably coronary atherosclerosis, had normal myocardial blood flow at rest but decreased coronary reserve in response to intracoronary adenosine in Doppler flow measurements [122] or to intravenous dipyridamole on PET [25, 30, 85, 164, 165]. Patients with chest pain had a more pronounced impairment of coronary reserve, [25] and the decrease in coronary reserve was related to poor clinical outcome. [30, 164] Intravenous infusion of the ACE inhibitor perindopilat in type 2 diabetic patients with LV hypertrophy improved the dipyridamole-recruitable coronary reserve acutely [85]. Patients with a genotype-positive sarcomeric mutation and hypertrophic cardiomyopathy had greater reduction in coronary reserve than genotype-negative patients, and they also had more fibrosis on gadolinium contrast MRI [165]. Multiparametric MRI appears to be of particular value in hypertrophic cardiomyopathy, as it can not only determine the severity of left ventricular hypertrophy and contractile dysfunction, but also the attenuation of coronary reserve and the extent of fibrosis [179]. As in the experimental studies, coronary blood flow is particularly impaired in patients with aortic stenosis when LV hypertrophy is associated with reduced coronary perfusion pressure [272]. On PET, the decrease in dipyridamole-recruitable coronary reserve was related to the severity of aortic stenosis and more pronounced in subendocardial than in subepicardial layers [188]. The magnitude of coronary reserve reduction was related to greater hypertrophy and left ventricular dysfunction and also to plasma hs-troponin T concentration as an injury marker, [282] and it was a marker of worse prognosis on follow-up [280]. The impairment of coronary reserve was reversible on transcatheter or surgical aortic valve replacement with regression of hypertrophy on follow-up [133, 282].

Hypertension not only induces LV hypertrophy but is also a major pathogenetic risk factor for coronary atherosclerosis; however, an impairment of dipyridamole-recruitable coronary vasodilator reserve is evident also in the absence of coronary artery disease [166, 204, 205, 225, 226]. The reduction in coronary reserve appeared to be specifically pronounced with hypertension as compared to other pathogenesis of left ventricular hypertrophy, [226] and a greater reduction in coronary reserve was associated with ST segment depression in Holter monitoring [205]. Episodes of ST segment depression corresponded to a greater reduction in subendocardial than subepicardial dipyridamole-recruitable coronary reserve in patients with hypertensive hypertrophy in PET [194]. Attenuation of coronary dilator reserve in patients with heart failure of hypertensive origin predicts worse clinical outcome on follow-up [281]. Chronic ACE inhibition with enalapril improved coronary reserve and reduced exercise-induced ST segment depression in a small group of hypertensive patients [151].

## Heart failure of ischemic and non-ischemic origin

### Heart failure with preserved ejection fraction

Heart failure with preserved ejection fraction is characterized by typical heart failure symptoms with mostly diastolic LV dysfunction but preserved ejection fraction. It is typically associated with comorbidities, such as obesity, diabetes and hypertension [182]. Experimental models of heart failure with preserved ejection fraction are available. With a more limited coronary microembolization than in the creation of heart failure with reduced ejection fraction, dogs developed heart failure with preserved ejection fraction [81]. In pigs with corticosterone-induced hypertension, heart failure with preserved ejection fraction developed and was characterized by decreased coronary reserve, [192] but no alteration in capillary density [152]. Pigs with chronic aortic banding developed LV hypertrophy with both systolic and diastolic dysfunction but still had preserved ejection fraction; [51] in this model, the increment in coronary blood flow for a given increase in myocardial oxygen consumption during pressure load was attenuated, suggesting impaired metabolic coronary vasodilation [51]. In a pig model with multiple comorbidities (diabetes, hyperlipidemia, renal hypertension), there was LV hypertrophy and fibrosis, but ejection fraction was preserved; [219] in this model there was increased nitric oxide synthase uncoupling, associated with increased reactive oxygen species formation and decreased nitric oxide bioavailability. Accordingly, the ex vivo coronary vasodilator responses to bradykinin were reduced [219]. In a mouse model of heart failure with preserved ejection fraction, secondary to a combination of hypertension through systemic nitric oxide synthase inhibition and a diet-induced obesity and metabolic syndrome, [206] there was an increased expression of inducible nitric oxide synthase with a resulting substantial increase in circulating nitric oxide which induced nitrosylation of proteins, including proteins of the unfolded protein response which serve to control protein quality. In this model, coronary endothelial function was impaired and coronary reserve was reduced [206].

In patients with heart failure and preserved ejection fraction, there is LV hypertrophy, fibrosis and microvascular coronary rarefication even in the absence of epicardial coronary stenosis at autopsy [150]. In the myocardium of these patients, there are an increased expression of inflammatory proteins as well as increased reactive oxygen species and decreased nitrite/nitrate concentrations secondary to increased vascular expression of NADPH oxidase and uncoupling of endothelial nitric oxide synthase [64]. Consistently, patients with heart failure and preserved ejection fraction have reduced coronary reserve in the absence of coronary artery disease [42, 114, 197, 222, 235] on Doppler angiography, [42, 197] PET [222, 235] or MRI [113, 114, 197]. Almost all patients with heart failure and preserved ejection fraction have either coronary artery disease on angiography, coronary microvascular dysfunction (increased minimal resistance on Doppler) and vasomotor dysfunction (impaired dilator response to acetylcholine) or both; [197] however, half of these patients have in fact epicardial coronary artery disease. The reduction in coronary reserve predicts adverse events on follow-up in these patients [113]. Collectively, coronary vascular dysfunction is a hallmark of heart failure with preserved ejection fraction, predisposing to myocardial ischemia. However, the causality of impaired coronary blood flow for the development of this heart failure entity is not established, as the typically predisposing comorbidities (obesity, diabetes, hypertension) each and in combination predispose also to coronary atherosclerosis such that heart failure with preserved ejection fraction and impaired coronary blood flow may develop in parallel from a common systemic inflammatory activation [175, 216].

### Takotsubo

Takotsubo cardiomyopathy is a clinical syndrome which is typically precipitated by extreme stress situation with an excessive catecholamine release [267] and characterized by features of both, myocardial infarction and heart failure [126]. Patients experience pain, ST segment alterations in their ECG and increased plasma troponin concentrations, mimicking acute myocardial infarction, yet their coronary circulation is not obstructed on angiography. Severe LV dysfunction with characteristic apical dyskinesis (“ballooning”) reflects the cardiomyopathy [70, 132, 177]. The takotsubo syndrome typically affects postmenopausal women in stress situations and it is reversible. The pathophysiology of the takotsubo syndrome is not fully clear, but coronary vascular dysfunction is definitely involved [260]. Using myocardial contrast echocardiography, a perfusion deficit in the dysfunctional region was identified [1, 67] which partially recovered during intravenous adenosine challenge along with an improvement of regional contractile function, [67] somewhat reminiscent of the Gregg effect seen in experimental studies of stunned myocardium [208]. Both, perfusion and contractile function recovered completely within 1-month follow-up [67]. On angiography, thrombolysis in myocardial infarction (TIMI) flow in patients with takotsubo was similarly impaired as in ST segment elevation myocardial infarction (STEMI) patients with microvascular obstruction on reperfusion [37]. Reduced myocardial blood flow reflecting coronary microvascular dysfunction was also demonstrated using single photon emission computed tomography [202, 270] and PET [32, 58, 121, 270] along with alterations in myocardial substrate metabolism suggestive of stunning/hibernation [58, 121, 202] and signs of inflammation [48, 267]. Endothelial dysfunction with focal or diffuse coronary vasoconstriction in response to intracoronary acetylcholine was seen a significant proportion of takotsubo patients [202]. While the pathophysiology of the takotsubo syndrome is not fully clear, the predominance of postmenopausal women being affected and the characteristic severe stress situations precipitating this syndrome suggest an interaction of estrogen deficiency possibly contributing to microvascular endothelial dysfunction [230] and increased responsiveness of the myocardium and coronary vasculature to catecholamines, which may be reflective of a more sparse sympathetic innervation of apical than basal myocardium [115] with a resulting catecholamine hypersensitivity [176]. Both, beta-adrenoceptor-mediated catecholamine toxicity on cardiomyocytes [132] and increased alpha-adrenoceptor-mediated vasoconstriction [95] may then induce a situation of transient ischemic dysfunction with subsequent stunning [132, 177]. However, at present, it is not fully clear whether reduced coronary blood flow is causal for the takotsubo syndrome; the only suggestive evidence originates from the observation that recruitment of dilator reserve with adenosine improves regional contractile function [67].

### Cardio-oncology

Patients with a cancer history have more coronary ischemic events [234] and a higher incidence of myocardial infarction [153] than those without. They also have a higher incidence of plaque erosion which is, in turn, associated with coronary microembolization, [117] and they have worse clinical outcome [234]. Cancer therapy not only induces toxic or inflammatory injury to cardiomyocytes [83, 241] but also to the vasculature, including the coronary vasculature [82, 178, 271]. Not only anti-angiogenic therapies, but also conventional chemotherapy or radiation therapy promotes reduced nitric oxide availability and endothelial dysfunction, predisposes to vasoconstriction and can ultimately precipitate angina or myocardial infarction. In a pig model of anthracycline cardiotoxicity, coronary arterial structural damage and reduced coronary reserve in response to papaverine became apparent before a myocardial contractile defect, whereas more microvascular structural alterations were only seen when also LV dysfunction had developed [66]. Whereas this study suggested that anthracycline chemotherapy-induced coronary vascular injury might contribute to LV dysfunction, the vascular and myocardial contribution to cardiac toxicity from chemotherapy and radiation therapy are clinically more difficult to dissect. Patients with pre-existing coronary artery disease have an increased risk to develop heart failure from anthracycline [54, 60, 184]. Thus, the contribution of an impaired coronary blood flow to the development of cancer therapy-induced heart failure is not really clear.

### The right ventricle in heart failure

The right ventricle is equally involved as the LV when the conditions causing heart failure affect the entire heart, such as genetic mutations, myocarditis, tachyarrhythmias or toxic agents, or when ischemia also affects right ventricular perfusion territories. The right ventricle may be less involved in failure when pressure or volume overload (hypertension, aortic valve disease) affects primarily the LV. The right ventricle, however, is more affected in pulmonary hypertension. The failing right ventricle has only recently received more attention, [79, 130, 261] and the coronary circulation in right ventricular failure has received little attention at all. Yet, there are some special considerations to the coronary circulation in the right ventricle, [35] since coronary perfusion pressure is above right ventricular pressure throughout the cardiac cycle such that extravascular compression and diastolic duration during tachycardia are of lesser importance than in the LV. Also, the thinner wall of the right ventricle may receive some retrograde perfusion through Thebesian veins. On the other hand, coronary autoregulation is less pronounced and alpha-adrenergic coronary vasoconstriction during sympathetic activation more pronounced in the right than the LV. Nevertheless, on the aggregate, the susceptibility to ischemia is less in the right than in the LV. However, in acute right ventricular pressure overload by acute pulmonary banding in dogs, there is increased alpha-adrenergic coronary vasoconstriction, increased extravascular compression and subendocardial ischemia [72, 73]. With chronic right ventricular pressure overload by chronic pulmonary stenosis, adenosine-recruitable coronary vasodilator reserve in the hypertrophied right ventricle is reduced particularly in the subendocardium [155] which impairs metabolic vasodilation during exercise [23, 156]. Patients with chronic pulmonary hypertension have reduced right coronary artery blood flow in proportion to right ventricular hypertrophy [257] and reduced adenosine-recruitable coronary reserve on MRI [262]. A recent NIH consensus workshop recommended directions for future research on the genetic, molecular and cellular processes in right heart failure, [130] but further research on the coronary circulation in right heart failure is also warranted. Arrhythmogenic right ventricular cardiomyopathy is a relatively infrequent form of human heart failure, caused by genetic mutations mostly in desmosomal proteins and characterized morphologically by diffuse fibrosis and inflammatory infiltration [68]. No specific alteration in coronary blood flow has been reported, but as in other heart failure entities, adenosine-recruitable coronary reserve on PET is reduced [174].

## Conclusions and directions for future research

Heart failure is almost invariably associated with coronary vascular dysfunction, not only in the frequent presence but also in the absence of coronary atherosclerosis. Cause-and-consequence relationships between heart failure and impaired coronary blood flow are complex. In stunning and hibernation, coronary microembolization, myocardial infarction and post-infarct remodeling, heart failure is clearly a consequence of myocardial ischemia without or with reperfusion—these are heart failure syndromes of ischemic origin. Vice versa, in all forms of heart failure, including hypertrophic and dilated cardiomyopathy with underlying genetic mutations and in the absence of coronary artery disease, increased extravascular compression and coronary vasoconstriction by the mediators of neurohumoral activation (norepinephrine, angiotensin, and endothelin) are clearly a consequence of heart failure. The invariably impaired endothelium-dependent coronary dilation as well as eventual morphological alterations of the coronary circulation could be a consequence of heart failure but also a consequence of the underlying conditions inducing heart failure (e.g., in pressure or volume overload). In some forms of heart failure, both ischemic and non-ischemic causes contribute to heart failure. In takotsubo cardiomyopathy, the causal contribution of coronary vascular and myocardial disturbances to the heart failure syndrome is not clear. In heart failure with preserved ejection fraction, the underlying comorbidities with the resulting systemic inflammatory state may cause both impairment of the coronary circulation and the myocardium in parallel. In aortic stenosis, there is both reduced coronary perfusion pressure causing ischemia and pressure overload causing LV hypertrophy.

**Fig. 5:**
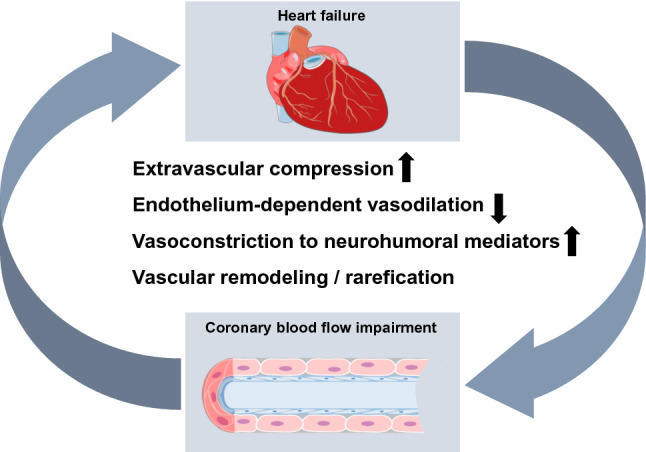
Vicious cycle between heart failure and impairment of coronary blood flow by common features of all heart failure entities: increased extravascular compression, reduced endothelium-dependent vasodilation, enhanced vasoconstriction to neurohumoral mediators and (to a variable extent) vascular remodeling and rarefication

In any form of heart failure, there is a vicious cycle between the impairment of myocardial contractile function and the impairment of the coronary circulation in that myocardial ischemia worsens heart failure and vice versa (Fig. 5), and it is reflected by the prediction of poor clinical outcome from heart failure by the reduction of coronary dilator reserve [116, 237].

Obviously, therapeutic restoration of coronary blood flow is of pivotal importance in all forms of heart failure for which ischemia is causal. For reversible ischemia and hibernating myocardium, the jury is still out in which clinical condition reperfusion by optimal medical therapy or by interventional/surgical revascularization is better. For irreversible ischemia and myocardial infarction, prevention of coronary microvascular obstruction is of pivotal importance. Unfortunately, interventional approaches using protection devices to attenuate coronary microvascular obstruction are of limited value and recommended only in cases of large atherothrombotic burden on angiography [117]. Also, pharmacological approaches to attenuate coronary microvascular obstruction, i.e., by use of adenosine, nitroprusside or calcium antagonists have been of limited clinical value [92, 96]. Currently, there is no evidence at all for clinical benefit from stimulation of angiogenesis through growth factor transfection or cell therapy. In heart failure of non-ischemic origin, there is no evidence that improvement of coronary blood flow specifically provides clinical benefit. Nevertheless, the above common features of coronary blood flow impairment in all forms of heart failure render them a valid target also for all established treatment strategies (statins, ACE inhibitors, AT1 blockers), but also a potential caveat (beta blockers: increased diastolic duration vs. increased vasoconstriction [86, 95]) and a worthwhile target in the study of novel treatment options, e.g., neprilysin or sodium glucose transporter 2 (SGLT2) inhibition. [6, 21, 143, 169, 181, 218].
